# Development of a Multiplex PCR Platform for the Rapid Detection of Bacteria, Antibiotic Resistance, and *Candida* in Human Blood Samples

**DOI:** 10.3389/fcimb.2019.00389

**Published:** 2019-11-13

**Authors:** Flora Marzia Liotti, Brunella Posteraro, Franca Mannu, Franco Carta, Antonella Pantaleo, Giulia De Angelis, Giulia Menchinelli, Teresa Spanu, Pier Luigi Fiori, Francesco Turrini, Maurizio Sanguinetti

**Affiliations:** ^1^Istituto di Microbiologia, Università Cattolica del Sacro Cuore, Rome, Italy; ^2^Istituto di Patologia Medica e Semeiotica Medica, Università Cattolica del Sacro Cuore, Rome, Italy; ^3^Dipartimento di Scienze Gastroenterologiche, Endocrino-Metaboliche e Nefro-Urologiche, Fondazione Policlinico Universitario A. Gemelli IRCCS, Rome, Italy; ^4^Nurex S.r.l., Sassari, Italy; ^5^Department of Biomedical Sciences, University of Sassari, Sassari, Italy; ^6^Dipartimento di Scienze di Laboratorio e Infettivologiche, Fondazione Policlinico Universitario A. Gemelli IRCCS, Rome, Italy; ^7^Department of Oncology, University of Turin, Turin, Italy

**Keywords:** multiplex PCR, bacteria, antibiotic resistance genes, *Candida*, blood samples, Microbscan assay

## Abstract

The diagnosis of bloodstream infections (BSIs) still relies on blood culture (BC), but low turnaround times may hinder the early initiation of an appropriate antimicrobial therapy, thus increasing the risk of infection-related death. We describe a direct and rapid multiplex PCR-based assay capable of detecting and identifying 16 bacterial and four *Candida* species, as well as three antibiotic-resistance determinants, in uncultured samples. Using whole-blood samples spiked with microorganisms at low densities, we found that the MicrobScan assay had a mean limit of detection of 15.1 ± 3.3 CFU of bacteria/*Candida* per ml of blood. When applied to positive BC samples, the assay allowed the sensitive and specific detection of BSI pathogens, including *bla*_KPC_-, *mecA*-, or *vanA*/*vanB*-positive bacteria. We evaluated the assay using prospectively collected blood samples from patients with suspected BSI. The sensitivity and specificity were 86.4 and 97.0%, respectively, among patients with positive BCs for the microorganisms targeted by the assay or patients fulfilling the criteria for infection. The mean times to positive or negative assay results were 5.3 ± 0.2 and 5.1 ± 0.1 h, respectively. Fifteen of 20 patients with MicrobScan assay-positive/BC-negative samples were receiving antimicrobial therapy. In conclusion, the MicrobScan assay is well suited to complement current diagnostic methods for BSIs.

## Introduction

Early diagnosis of bloodstream infections (BSIs), including those caused by bacteria and fungi, is central to reducing drastic infection-related consequences, such as significant risks of morbidity and mortality (Goto and Al-Hasan, 2013; Andes et al., 2016; Seymour et al., 2017). Global estimates show that in high-income countries, the incidence rates for sepsis and severe sepsis cases were 437 and 270 per 100,000 person-years, respectively, with hospital mortality rates of 17.0% for sepsis and 26.0% for severe sepsis during the last decade (Fleischmann et al., 2016). In the United States, the incidence of patients receiving treatment for septic shock rose from 12.8 to 18.6 cases per 1,000 hospitalizations over 10 years, while mortality declined from 54.9 to 50.7% (Kadri et al., 2017). In a recent study, a delay of 50.0 min for blood culture (BC) or 125.0 min for antibiotic therapy within the 3-h Surviving Sepsis Campaign guideline increased the risk of death in patients with severe sepsis and septic shock (Pruinelli et al., 2018).

The reference standard for the microbiological diagnosis of a BSI is still an automated BC system (Baron et al., 2013). However, despite the good analytical sensitivity of the BC method (limit of detection [LOD], 1–10 CFU/ml) (Yagupsky and Nolte, 1990; Pfeiffer et al., 2011), slow turnaround time (TAT) is the main limitation (Lamy et al., 2016), which necessitates the aggressive empirical usage of antimicrobial agents (Banerjee et al., 2016; Timbrook et al., 2017). Furthermore, delays in optimal antimicrobial therapy may drive poor health care quality as well as increase antimicrobial resistance (Zasowski et al., 2016; Whiles et al., 2017). Early initiation and modification of appropriate antimicrobial therapy depends on the availability of diagnostic methods with rapid TATs, e.g., within 6–8 h of septic patient presentation (Ginn et al., 2017). Molecular detection methods applied to positive BCs or direct blood samples show promise in accelerating microbial identification and predicting antimicrobial susceptibility (Dubourg and Raoult, 2016; Peker et al., 2018). It is imperative that diagnostic tests are tailored to the clinical problem at hand to maximize the cost-effectiveness of clinical decision-making (Pliakos et al., 2018).

Commercially available methods performed directly on whole-blood samples include nucleic amplification-based methods and, only recently, T2 magnetic resonance-based methods (Peker et al., 2018). Alternatively, a plasma metagenomics-sequencing assay (Karius, Redwood City, CA) generates sequences of circulating microbial cell-free DNA to enable the non-invasive diagnosis of infectious diseases (Blauwkamp et al., 2019). Compared to post-culture detection (e.g., by FilmArray^®^ Blood Culture Identification Panel; bioMérieux, Marcy-l'Étoile, France), direct detection (from blood) eludes the “test method-related” shortcomings that may lead to false-negative diagnoses because of slow or not growing microorganisms and cases when the patient has already received antimicrobials (Farrell et al., 2013). For example, the LightCycler^®^ SeptiFast (Roche Molecular System, Switzerland; www.molecular.roche.com) is a Conformité Européenne (CE)-marked multiplex real-time PCR (qPCR) assay that simultaneously detects and identifies DNA from 19 bacterial and 6 fungal species. In a subsequent run, the test assesses the presence of methicillin resistance in *Staphylococcus aureus*-positive samples (Peker et al., 2018). Importantly, the recent finding of significant rates of false positive (up to 20% or higher) and false negative (up to 14%) results has limited the utility of the LightCycler^®^ SeptiFast as a standalone test (Ginn et al., 2017). However, multiplex qPCR assays may offer several advantages (e.g., high-throughput and quantification) (Bustin et al., 2009), making them well suited to the clinical setting (Farrell et al., 2013).

**Figure 1:**
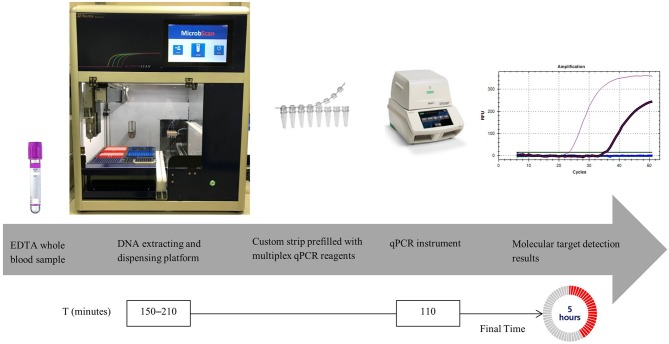
MicrobScan assay workflow.

We developed a multiplex qPCR-based detection molecular assay (hereafter designated the MicrobScan assay) that can process a wide range of microorganism densities (CFU/ml) directly from blood samples (Figure 1). Specifically, we tested the general capabilities of this assay using low-density spikes of phenotypically characterized and culture-quantified bacterial or *Candida* species in blood. By processing samples from a positive blood culture bottle (PBCB) without measuring the density, we show that the assay allows the sensitive and specific identification of 20 frequently encountered microbial pathogens and relevant antimicrobial resistance genes. Finally, we evaluated the clinical performance of the assay with blinded whole-blood samples from patients with suspected BSI.

## Materials and Methods

### Description of the MicrobScan Assay

We designed the MicrobScan assay to run on a fully automated SepsiScan instrument (Nurex S.r.l., Sassari, Italy), where a robot can handle 24 samples in parallel. The assay requires minimal hands-on time (e.g., typically <15 min) from putting the sample into a microwell strip to loading the strip onto the BioRad CFX Thermal Cycler (BioRad, Hercules, CA, USA), which is a PCR system for gene amplification. We installed the SepsiScan instrument at the clinical microbiology laboratory of the Fondazione Policlinico Universitario A. Gemelli IRCCS of Rome (Italy), where it automatically completed all assay steps in prefilled wells starting from 100 μl of the original sample (i.e., EDTA whole blood). Specifically, the procedural steps that are automated by the SepsiScan instrument are as follows: detergent lysis of blood cells; concentration of cellular debris and microbial cells; extraction of microbial DNA for use as a template in the multiplex qPCRs (see below); and finally bleach decontamination of all liquids on the SepsiScan instrument rack. In particular, DNA extraction is achieved through the sequential action of lytic enzymes (lysozyme, lyticase, chitinase, and proteinase K) and strong detergents (including sodium dodecyl sulfate) and the subsequent purification by binding the extracted DNA to magnetic beads. After alternate guanidine thiocyanate/ethanol washing, microbial DNA is eluted from the beads in 150-μl elution buffer (Tris-EDTA plus 1% Triton X-100), concentrated in a microfluidic extraction cartridge, and immediately dispensed in an 8-well strip containing dried PCR reagents. The entire process enables users to avoid any contamination by hemoglobin, iron, and any blood-cell PCR inhibitor. The assay allows for the simultaneous detection and identification of 20 bacterial/*Candida* species and 3 antibiotic resistance-associated genes. We chose the 20 microorganisms, with or without any antibiotic-resistance determinant, as molecular targets for the MicrobScan assay based on their prevalence in clinical laboratory BC assays. The MicrobScan assay has recently received CE marking.

### Growth and Quantification of Microorganisms

The microorganisms used in this study were well-characterized bacteria and *Candida* strains obtained from the ATCC^®^ (Manassas, VA, USA) and/or clinical isolates obtained from the aforementioned clinical microbiology laboratory. In addition to clinical isolates (*N* = 333), the ATCC^®^ strains included *Acinetobacter baumannii* (19606™), *Aspergillus fumigatus* (13073™), *Bacteroides fragilis* (25285™), *Burkholderia cepacia* (25416™), *Candida albicans* (10231™), *Candida glabrata* (2001™), *Candida krusei* (6258™), *Candida orthopsilosis* (20503™), *Candida parapsilosis* (7330™), *Candida tropicalis* (13803™), *Corynebacterium striatum* (BAA-1293™), *Corynebacterium ramosum* (25582™), *Cryptococcus neoformans* (32045™), *Enterobacter aerogenes* (13048™), *Enterobacter cloacae* (13047™), *Escherichia coli* (25922™, BAA-2340™), *Enterococcus faecalis* (29212™, 51299™), *Enterococcus faecium* (55593™, 700221™), *Haemophilus influenzae* (9006™), *Klebsiella oxytoca* (700324™), *Klebsiella ozaenae* (25926™), *Klebsiella pneumoniae* (13883™, BAA-1705™), *Legionella pneumophila* (33152™), *Moraxella catarrhalis* (25238™), *Neisseria meningitidis* (13098™), *Pseudomonas aeruginosa* (27853™), *Proteus mirabilis* (7002™), *Proteus vulgaris* (13315™), *Serratia marcescens* (8100™), *S. aureus* (29213™, 43300™), *Staphylococcus epidermidis* (12228™, 35984™), *Staphylococcus haemolyticus* (29970™), *Staphylococcus hominis* (700586™), *Stenotrophomonas maltophilia* (13637™), *Streptococcus agalactiae* (12403™), *Streptococcus pneumoniae* (6305™), *Streptococcus pyogenes* (12344™), *Streptococcus parasanguinis* (31412™), and *Streptococcus salivarius* (25975™). We grew all the microbes on appropriate agar media to harvest single colonies, which were diluted 10-fold at various concentrations in sterile phosphate-buffered saline and then quantified by culture onto the media to estimate the CFU per milliliter.

### Primer and Taqman Probe Selection

We detected 20 clinically relevant microorganisms using species- or group-specific target genes as follows: *bap* gene for *A. baumannii* (De Gregorio et al., 2015); *leuB* gene for *B. fragilis* (Papaparaskevas et al., 2013); 5.8S rRNA gene for *C. albicans*; internal transcribed spacer 1 (ITS1), 5.8S rRNA, and ITS2 genes for *C. glabrata* and *C. krusei* (Metwally et al., 2007); 18S rRNA gene for *Candida* spp.; *omp35* gene for *E*. aerogenes (van der Zee et al., 2016); *apbC* gene (formerly referred to as unknown gene) for *E. cloacae* complex (van der Zee et al., 2016); 16S rRNA gene for *E. coli* (van den Brand et al., 2014); *groESL* gene for *E. faecalis* and *E. faecium* (Fukumoto et al., 2015); *rhaA*-*rhaD* operon gene for *Klebsiella* spp. (van den Brand et al., 2014); *phzE* gene for *P. aeruginosa* (van den Brand et al., 2014); *hns* gene for *P. mirabilis* (van der Zee et al., 2016); *gyrB* gene for *S. marcescens* (van den Brand et al., 2014); *spa* gene for *S. aureus*; *tuf* gene for *Staphylococcus* spp. (van den Brand et al., 2014); *lytA* gene for *S. pneumoniae* (Gadsby et al., 2015); *sdaB* gene for *S. pyogenes* (Fukumoto et al., 2015); and the 16S rRNA gene for *Streptococcus* species. Additionally, we detected three antibiotic-resistance genes, including the *bla*_KPC_ carbapenemase gene (Zheng et al., 2013), the *mecA* methicillin resistance gene (Thomas et al., 2007), and the *vanA/vanB* vancomycin resistance genes. Finally, we used the human RNase gene as an internal control for sample DNA. Unless otherwise specified, we selected primer pairs and Taqman probes from conserved regions of the respective specific sequences using Beacon Designer™ software (Premier Biosoft, Palo Alto, CA, USA). To test for any potential cross-reactivity, we performed *in silico* specificity studies to compare the oligonucleotide sequences to all known genetic sequences.

### Multiplex qPCR System

The MicrobScan assay consists of seven parallel qPCRs in seven reaction wells, including 1 duplex, 3 triplex, and 3 quadruplex reaction wells, which target the 20 microorganisms and 3 antibiotic-resistance determinants per sample. Another well serves as an RNase gene-based control. We optimized the qPCRs by testing different concentrations of each primer pair and Taqman probe, as well as buffer components, and we calculated PCR efficiencies from the slopes of standard curves that were run in triplicate (data not shown). The final 30-μl qPCR mixture contained deoxynucleoside triphosphates (including dUTP) and uracil-N-glycosylase. We used concentrations of 400 nM for all primer pairs and 150 nM for all probes. We carried out qPCRs using the aforementioned BioRad CFX Thermal Cycler using the following thermal cycling conditions: an initial denaturation step at 95°C for 10 min, followed by 40 cycles of 94°C for 15 s and 60°C for 30 s. We ran positive (DNA mixtures of all the targets) and negative (sterile water) PCR controls in qPCRs for each test sample batch. We analyzed qPCR data using proprietary software (Nurex S.r.l.) designed *ad hoc* to generate a quantification cycle (Cq) (formerly referred to as threshold cycle [Ct]) (Bustin et al., 2009) value for each of the targets. Based on our experiments with spike-in controls, a Cq value of 40 was set up as a cutoff value for positivity, and we considered a sample as positive if the Cq value was ≤40. We based the differentiation between *S. aureus* and one *Staphylococcus* species, between *S. pneumoniae*/*S. pyogenes* and one *Streptococcus* species and between *C. albicans* and one *Candida* species on the results of two corresponding qPCRs, respectively. If both qPCRs were positive, we identified the microorganism(s) as belonging to the specific species, whereas if only the species-group (i.e., *Staphylococcus* spp., *Streptococcus* spp., or *Candida* spp.) relative qPCR was positive we identified the microorganism(s) as belonging to the specific genus group.

### Analytical Studies

We determined the LOD for the assay on isolates from 18 species of bacteria and 6 species of *Candida*, including species representative of *Staphylococcus* spp. other than *S. aureus, Streptococcus* spp. other than *S. pneumoniae* and *S. pyogenes*, and *Candida* spp., using quantified spiked samples. We prepared the sample matrix for spiking experiments using fresh microbial suspensions to the appropriate density (CFU/ml) to obtain replicate samples for testing. Then, we performed a Probit analysis, which transforms the proportions of positive results detected into a “probability unit” (or “probit”), using the Analyze-it statistical analysis software addendum for Microsoft Excel (Analyze-it Software Ltd., Leeds, UK). Briefly, we used several dilutions of the microbial suspensions (CFU/ml) to prepare eight replicates for each density. We plotted the probit (y-axis) against the logarithm of the density (x-axis), and we calculated the 95% LOD value, which was the density of each microbial species in a sample that yielded positive detection 95% of the time. We assessed the assay reproducibility (also known as variability) using the same replicate experiments performed to determine LOD, and we calculated the mean Cq values for both target and internal control, along with the coefficient of variation (% CV) as a variance measure. Finally, we performed a carryover assessment by testing adjacent negative and highly positive samples. Positives consisted of samples spiked with 10^6^ or 10^7^ CFU/ml of either *bla*_KPC_-positive *Klebsiella pneumoniae* [KPC], *vanA*/*vanB*-positive *Enterococcus faecium*/*Enterococcus faecalis* [VRE]), or both *mecA*-positive *S. aureus* [MRSA] and *Candida albicans*.

### Spiked and Clinical Blood Samples

We collected healthy volunteer blood samples, and we made final dilutions of each of the aforementioned microorganisms for generating blood samples spiked with cultured bacteria/*Candida* species at a range of densities (10^7^-10^1^ CFU/ml). These samples served to either optimize the amount of blood for clinical testing or determine the LOD for each MicrobScan assay target (as specified above), as well as to exclude any between-target cross-reactivity. We used a set of 87 non-consecutive fresh positive BCs (BacT/ALERT^®^ FA, FN, and PF Plus bottles, bioMérieux, Marcy l'Étoile, France) obtained during routine clinical microbiology at the Fondazione Policlinico Universitario A. Gemelli IRCCS of Rome (Italy). These samples were used to determine the analytical sensitivity and specificity of the MicrobScan assay regardless of the density (CFU/ml) of microorganisms cultured from the blood. Additionally, we collected 229 EDTA whole blood (2–3 ml) samples drawn from prospectively consenting patients from September 2017 to August 2018 at the aforementioned Fondazione Policlinico Universitario A. Gemelli IRCCS, using the same venipuncture used for the BC samples. These samples were from patients for whom physicians ordered BCs, as a part of the standard of care per hospital protocol, due to the clinical suspicion of a BSI. Each whole blood sample was used to assess the clinical sensitivity and specificity of the MicrobScan assay; all samples were refrigerated at 4°C within 30 min of collection and then frozen until analysis.

### Microbiological Methods

We performed culture-based identification and antimicrobial susceptibility testing of BSI isolates according to standard laboratory procedures (Baron et al., 2013). Briefly, we incubated all patient BC bottles that arrived at the clinical microbiology laboratory in the BacT/Alert^®^ Virtuo^®^ system (bioMérieux) at 37°C for up to 5 days or until they signaled a positive result. At the time bottles gave a positive signal or at the end of their incubation period, we performed subcultures of the BC medium on blood (for bacteria) or Sabouraud dextrose (for yeast) agar plates to assess true-positive and true-negative detections. We identified the bacteria and *Candida* isolates grown in the blood by the MALDI BioTyper^®^ system (Bruker Daltonics, Bremen, Germany) following the manufacturer's instructions. Then, we tested bacterial isolates for antimicrobial susceptibility using the VITEK 2^®^ automated system (bioMérieux), and we used the 2017 EUCAST standards to interpret antimicrobial susceptibility testing (AST) results (http://www.eucast.org/fileadmin/src/media/PDFs/EUCAST_files/Breakpoint_tables/v_7.1_Breakpoint_Tables.pdf). For bacterial isolates found to be phenotypically resistant to extended-spectrum beta-lactamases, oxacillin, or vancomycin, we performed PCR amplification of the isolate DNA as previously described (Fiori et al., 2016) to confirm the presence of *bla*_KPC_, *mecA*, or *vanA*/*vanB* genes, respectively, in these isolates.

### Data Analysis

We initially estimated the diagnostic performance of the MicrobScan assay by calculating sensitivity, specificity, negative predictive value (NPV), and positive predictive value (PPV) compared to the BC method. To resolve discrepancies between the MicrobScan assay and BC results, we used a synopsis of clinical and/or laboratory data to identify the “true-infection” cases among those that yielded false-positive MicrobScan assay results. These data included microbiological results of BCs from other BSI episodes and of cultures from other sites of infection that were available within ± 7 days from the index positive BC and consultation with the treating physician. Based on this composite diagnostic criterion, we calculated the overall agreement between the MicrobScan assay and BC results. Finally, we compared the time to positive detection by the MicrobScan assay with the time to culture-based species identification (i.e., the time to BC positivity plus the time to result by standard identification). Additionally, we used antimicrobial therapy information to assess the potential influence of prior antimicrobial use on the MicrobScan assay performance as well as the potential impact of the MicrobScan assay results on the antimicrobial treatment changes (i.e., escalation or de-escalation) (Murri et al., 2018) in patient subgroups. We performed statistical analyses using IBM SPSS software version 24.0 (Armonk, NY, USA), and we compared continuous variables (expressed as the means ± SD) using Student's *t*-test and categorical variables (expressed as counts and percentages) using Fisher's exact test. Differences were considered to be statistically significant at *P* < 0.05.

## Results

### Analytical Sensitivity

We used qPCR primers designed to amplify unique sequences in species-specific bacterial or *Candida* genes and in *bla*_KPC_, *mecA*, or *vanA*/*vanB* antibiotic resistance genes representing 23 molecular targets in the MicrobScan assay (Table 1). We combined multiple primers for the simultaneous recognition of two, three, or four of the 23 targets in seven single-reaction (one duplex, three triplex, and three quadruplex) wells. This *in silico*-generated format allowed multiple fluorescently labeled TaqMan probes to react with each of the corresponding PCR amplicons in the well. First, we measured the LOD of the MicrobScan assay with all targeted microorganisms (including KPC, MRSA, and VRE) in both sterile phosphate-buffered saline and whole blood matrices by means of singleplex qPCRs. All microorganism LODs were similar between these two matrices, and the PCR efficiency was >97% for each microorganism (see Table S1). The assay had a mean (± SD) LOD of 15.6 ± 3.1 CFU for bacteria (13.3 ± 1.6 for gram-negative and 18.5 ± 1.9 for gram-positive) or *Candida* (16.5 ± 2.0) per ml of blood, and all targeted microorganisms yielded LODs between 10 and 21 CFU/ml (see Table S1). All the targets amplified with a Cq value between 22 and 38. The calibration curve indicated a linear dynamic range of 10^6^-10^1^ CFU/ml. The primer pairs and probes in multiple reactions did not significantly affect the sensitivity compared with that of singleplex reactions. The assay variation during the detection and identification of 3 × LOD spikes of the test microorganisms ranged from +0.4 to +1.1 Cq values. Next, we characterized the repeatability of the MicrobScan assay by testing microorganisms at either 3 × LOD or 10 × LOD concentrations. We spiked all the microorganisms into whole blood, and the observed % CV among replicates was <30%, whereas the mean Cq varied significantly depending on the 3 × LOD or 10 × LOD concentration. Finally, among the independent possibilities to observe carryover events based on the assay configuration, we did not observe any carryover event.

**Table 1:** Sequences of primers and probes used in the multiplex real-time PCRs.

Species^*^	**Target gene**	**Primer name**	**Primer sequence (5'−3')**	**Probe name**	**Probe sequence (5′-3′)^†^**	**Amplicon size (bp)**	**Reference**
**GRAM-NEGATIVE SPECIES**							
*Acinetobacter baumannii*	*bap*	Aba-f	CGCTGCAGCATCAAATCATG	Aba-Pro	Cy5-AGCACCTGCTGACACCACTCCACCA-BHQ2	205	De Gregorio et al., 2015
		Aba-r	TGGGTCAACCGAGAAAGTTACG				
*Bacteroides fragilis*	*leuB*	Baf-f	CACTTGACTGTTGTAGATAAAGC	Baf-Pro	FAM-TGTGCTTGCTTCCAGTCGTCTATG-BHQ1	135	Papaparaskevas et al., 2013
		Baf-r	CATCTTCATTGCAGCATTATCC				
*Enterobacter aerogenes*	*omp35*	Ena-f	CCCATGCTTCAGCTTTGTCA	Ena-Pro	TxRd-CGTTGCCGTCACGTTTCTGGTCAA-BHQ2	74	van der Zee et al., 2016
		Ena-r	CTGCAGGTTACGCTAACTCCAA				
*Enterobacter cloacae* complex	*apbC*	Enc-f	ACAAAGGAGTCGGGATGAGTTC	Enc-Pro	FAM-CAATCCCAGGCCAAATCACCGG-BHQ1	65	van der Zee et al., 2016
		Enc-r	CGACCATTGCTCGTAAGGCT				
*Escherichia coli*	16S rRNA	Eco-f	CATGCCGCGTGTATGAAGAA	Eco-Pro	FAM-TATTAACTTTACTCCCTTCCTCCCCGCTGAA-BHQ1	96	van den Brand et al., 2014
		Eco-r	CGGGTAACGTCAATGAGCAAA				
*Klebsiella* spp.	*rhaA*–*rhaD* operon	Kle-f	AACCAGGCGTCGATAAT	Kle-Pro	HEX-ACAGGAAAGACAAGACTATGCAGACC-BHQ1	107	van den Brand et al., 2014
		Kle-r	GTTTACGGCGCAATCC				
*Proteus mirabilis*	*hns*	Pmi-f	GCACGTTTAGCACGACCAGTT	Pmi-Pro	TxRd-CGCCAGCAGCTTCAAGCAGGTCA-BHQ2	71	van der Zee et al., 2016
		Pmi-r	TGCCGATGGTATTGATCCAA				
*Pseudomonas aeruginosa*	*phzE*	Pae-f	GCCGAGGTCATGGAATTC	Pae-Pro	HEX-CGACAACCGCAAGGAAGCCGA-BHQ1	89	van den Brand et al., 2014
		Pae-r	ATCCGCGCCATCATCTTC				
*Serratia marcescens*	*gyrB*	Sma-f	GACCGTGAAGACCACTTCCATTAC	Sma-Pro	Cy5-CGATCCACCCGAACGTGTTCTACTTCTC-BHQ2	125	van den Brand et al., 2014
		Sma-r	ACGCCGATGTCGTCTTTCAC				
**GRAM-POSITIVE SPECIES**							
*Enterococcus faecalis*	*groESL*	Efs-f	TGATGCCCGCGTTCATTTA	Efs-Pro	Cy5-AAACCAAATCGGCGAAACAACGTCTG-BHQ2	77	Fukumoto et al., 2015
		Efs-r	CGTTCTTGTAATTTTTCACGATCAA				
*Enterococcus faecium*	*groESL*	Efm-f	GTATCAAGATCGTTGTTCGTGCTT	Efm-Pro	TxRd-AGAAGAACCAATTCGTCAAATCGCTG-BHQ2	74	Fukumoto et al., 2015
		Efm-r	CTGATCCTTCATAACCAGCGTTT				
*Staphylococcus aureus*	*spa*	Sau-f	CAGCAAACCATGCAGATGCTA	Sau-Pro	FAM-AAAGCTCAAGCATTACCAGAAACTG-BHQ1	101	This study
		Sau-r	CGCTAATGATAATCCACCAAATACA				
*Staphylococcus* spp.	*tuf*	Sta-f	CCAACWCCAGAACGTGAYTCTG	Sta-Pro	HEX-ACAGGCCGTGTTGAACGTGGKCAAATCAA-BHQ1	222	van den Brand et al., 2014
		Sta-r	GTTRTCACCAGCTTCAGCGTART				
*Streptococcus pneumoniae*	*lytA*	Spn-f	ACGCAATCTAGCAGATGAAGCA	Spn-Pro	HEX-TGCCGAAAACGCTTGATACAGGGAG-BHQ1	74	Gadsby et al., 2015
		Spn-r	TCGTGCGTTTTAATTCCAGCT				
*Streptococcus pyogenes*	*sdaB*	Spy-f	GGRACACGTACCCAAAATGTAGGA	Spy-Pro	TxRd-CGTGACCAAAAAGGCGGCATGC-BHQ2	73	Fukumoto et al., 2015
		Spy-r	TCTTGAGCTCTTTGTTCGGTRTAG				
*Streptococcus* spp.	16S rRNA	Str-f	ATCCTTWCTAAAGAAGAAG	Str-Pro	FAM-CTCCATTCTTCAACAACTACCG-BHQ1	117	This study
		Str-r	GTTATCACCAGGCATWAC				
**FUNGAL SPECIES**							
*Candida albicans*	5.8S rRNA	Calb-f	GGTTTGCTTGAAAGACGGTA	Calb-Pro	Cy5-TTACCGCCGCAAGCAATGTT-BHQ2	109	This study
		Calb-r	AGTTTGAAGATATACGTGGTA				
*Candida krusei*	ITS1, 5.8S rRNA, ITS2	Ckru-f	CCTGTTTGAGCGTCATTTCC	Ckru-Pro	HEX-AGCTGGCCGAGCGAACTAGACTTTT-BHQ1	219	Metwally et al., 2007
		Ckru-r	CCTGCTTTGAACACTCTAA				
*Candida glabrata*	ITS1, 5.8S rRNA, ITS2	Cgla-f	CCTGTTTGAGCGTCATTTCC	Cgla-Pro	FAM-TAGGTTTTACCAACTCGGTGTTGAT-BHQ1	229	Metwally et al., 2007
		Cgla-r	AGCACGCACAAAACACTCACTTAT				
*Candida* spp.	18S rRNA	Can-f	TAGTTGGTGGAGTGATTTGTCTG	Can-Pro	TxRd-AACCTACTAAATAGTGCTGCTAGCCAT-BHQ2	159	This study
		Can-r	TAAGGGCATCACAGACCTGTTA				
**ANTIMICROBIAL-RESISTANT SPECIES**							
Carbapenem-resistant	*bla*_KPC_	Kpc-f	CGCAACTGTAAGTTACCG	Kpc-Pro	Cy5-CCACTGTGCAGCTCATTCAAGG-BHQ2	187	Zheng et al., 2013
		Kpc-r	CATGCCTGTTGTCAGATA				
Methicillin-resistant	*mecA*	Mec-f	AAAGAACCTCTGCTCAACAAGT	Mec-Pro	HEX-CCAGATTACAACTTCACCAGGTTCAACT-BHQ1	88	Thomas et al., 2007
		Mec-r	TGTTATTTAACCCAATCATTGCTGTT				
Vancomycin-resistant	*vanA*	VanA-f	TCTTAATTGAGCAGGCTGT	VanA-Pro	FAM-TACCGCACAACCGACCTCACA-BHQ1	82	This study
		VanA-r	CACCTCGCCAACAACTA				
	*vanB*	VanB-f	GATAGAAGCAGCAGGACAAT	VanB-Pro	FAM-CGCAGCCGACCTCACAGC-BHQ1	109	This study
		VanB-r	CGCCGACAATCAAATCATC				

*Duplex, triplex, or quadruplex reactions were optimized to include Taqman probes, together with each relative primer (forward [f] and reverse [r]) pair, in single wells, which allowed the detection of the DNA from two, three, or four of the 23 listed molecular targets. The best combinations were Staphylococcus aureus and Staphylococcus spp.; bla_KPC_, mecA, and vanA/vanB; Klebsiella aerogenes, Enterobacter cloacae complex, and Klebsiella spp.; Enterococcus faecalis, Enterococcus faecium, and Streptococcus spp.; Acinetobacter baumannii, Escherichia coli, Pseudomonas aeruginosa, and Proteus mirabilis; Bacteroides fragilis, Serratia marcescens, Streptococcus pyogenes, and Streptococcus pneumoniae; Candida albicans, Candida glabrata, Candida krusei, and Candida spp. As a result, the MicrobScan assay consisted of eight distinct wells (seven with multiplex organism-specific reactions and one with a human RNase-coding gene-based control) per sample*.

* *Including Klebsiella pneumoniae, Klebsiella oxytoca, and Klebsiella ozaenae (Klebsiella spp.); S. aureus, Staphylococcus epidermidis, Staphylococcus hominis, Staphylococcus haemolyticus, Staphylococcus lugdunensis, and Staphylococcus warneri (Staphylococcus spp.); Streptococcus agalactiae, Streptococcus anginosus, Streptococcus gallolyticus, Streptococcus gordonii, Streptococcus oralis, Streptococcus mitis, Streptococcus parasanguinis, Streptococcus pneumoniae, and Streptococcus pyogenes (Streptococcus spp.); and Candida albicans, Candida parapsilosis, Candida orthopsilosis, and Candida tropicalis (Candida spp.)*.

† *All probes were labeled with a reporter dye (i.e., cyanine5 [Cy5], 6-carboxyfluorescein [FAM], hexachlorofluorescein [HEX], or Texas Red [TxRd]) at the 5'-end and with a quencher (i.e., Black Hole Quencher 1 [BHQ1] or Black Hole Quencher 2 [BHQ2]) at the 3'-end*.

### Analytical Specificity

We evaluated the specificity of the multiplex qPCRs for the identification of 20 microbes at the species level (including *Klebsiella* spp., *Staphylococcus* spp., *Streptococcus* spp., and *Candida* spp.) and for the detection of three (carbapenem, methicillin, or vancomycin) resistance determinants. As shown in Table S2, each of the six species-specific qPCRs correctly detected DNA from the corresponding microbial species with 100% accuracy; the PCRs did not detect DNA from reference ATCC strains or clinical isolates belonging to the different species targeted or non-targeted by the MicrobScan assay. The latter species comprised *A. fumigatus, B. cepacia, C. striatum, C. ramosum, C. neoformans, H. influenzae, L. pneumophila, M. catarrhalis, N. meningitidis, P. vulgaris*, and *S. maltophilia*. Each resistance determinant-specific qPCR showed positive results only with *K. pneumoniae* (ATCC^®^ BAA-1705™), *S. aureus* (ATCC^®^ 43300™), *E. faecium* (ATCC^®^ 700221™), and *E. faecalis* (ATCC^®^ 51299™) strains, which harbored the *bla*_KPC_, *mecA, vanA*, or *vanB* genes, respectively. We did not observe amplification with human DNA except in the sample control (RNase-specific) well. Based on these findings, the MicrobScan assay developed here had 100% specificity.

### Direct Detection of Microbial Pathogens From PBCB Samples

In the routine molecular diagnosis of BSIs, we and other clinical microbiologists currently use PBCBs, as testing these samples shortens the time to microbial species identification (Fiori et al., 2014). We applied the MicrobScan assay to 87 samples consisting of aliquots of PBCBs, which yielded isolates in mono- (*N* = 61) and polymicrobial (*N* = 26) growth (Tables 2, 3). For monomicrobial cultures, we obtained 100% detection matching the species identified by culture, except for one sample positive for *B. cepacia* that the assay was not designed to detect (Table 2). For the polymicrobial cultures, the presence of multiple microorganisms did not prevent the MicrobScan assay from detecting and identifying at least one of the components of the mixed culture. Except for two samples positive for *C. striatum* and *C. ramosum* that the assay was not designed to detect, 18 (75.0%) of 24 samples had the second and two of 18 had the third microorganism detected by the MicrobScan assay (Table 3). While differences in the Cq for single microorganism detection reflected differences in the CFU/ml of single microorganisms concomitantly present in a PBCB sample (i.e., a lower Cq corresponded to a higher CFU/ml), failures to detect the second microorganism in 6 (25.0%) of 24 samples did not always reflect differences in the CFU/ml between concomitant microorganisms. Expectedly, failures often occurred in samples containing two (or three) microorganisms from species that the assay detects in the same reaction well (see Table S2). One example was *A. baumannii* (CFU/ml, 2 × 10^8^) that grew in culture together with *P. aeruginosa* (1 × 10^6^ CFU/ml), which the assay failed to detect (Table 3). Another example was *S. epidermidis* (1.5 × 10^8^ CFU/ml) that grew in culture together with *A. baumannii* (1 × 10^6^ CFU/ml), which the assay also failed to detect (Table 3). In one case, PCR primers and probes for *A. baumannii* and *P. aeruginosa* worked in the same reaction; in the other case, the primers for *A. baumannii* and *S. epidermidis* did not work in the same reaction (see Table S2). Interestingly, in two samples both positive for *S. aureus* and a *Staphylococcus* species other than *S. aureus* (*S. capitis* and *S. epidermidis*, respectively), the species-specific probes yielded two sharp fluorescence signals that strongly suggested the concomitant presence of two staphylococci. These samples were clearly distinguished from the samples only containing *S. aureus* that, indeed, gave a strong signal with the *S. aureus*-specific probe and a weak signal with the probe specific for *Staphylococcus* species other than *S. aureus*. Additionally, both the laboratory reference method and the MicrobScan assay detected a microorganism associated with one of the antibiotic resistance determinants targeted by the MicrobScan assay. Thus, in 100% of samples (28/28), the reference method results and those of the MicrobScan assay were fully concordant (9/9 *bla*_KPC_, 18/18 *mecA*, and 1/1 *vanA*/*vanB*). Of 18 *mecA* genes detected, 12 were from *S. aureus*, and six were from coagulase-negative staphylococci (5 *S. epidermidis* and 1 *S. hominis*).

**Table 2:** Results for 61 positive blood culture broth (PBCB) samples with monomicrobial growth tested by the MicrobScan assay.

**PBCB testing**	**MicrobScan assay testing**	
**Species identified (no. of isolates)**	**Positive**	**Cq**
*Acinetobacter baumannii* (2)	2/2	17.1 ± 0.5
Burkholderia cepacia^*^ (1)	0/1	–
*Candida albicans* (1)	1/1	18.7
*Candida glabrata* (1)	1/1	18.8
*Candida* species (2)	2/2	18.9 ± 1.7
Coagulase-negative staphylococci (4)^†^	4/4	17.2 ± 2.1
*Enterobacter cloacae* (1)	1/1	18.3
*Enterococcus faecalis* (2)	2/2	12.5 ± 0.2
*Enterococcus faecium* (3)	3/3	14.3 ± 0.4
*Escherichia coli* (11)	11/11	17.9 ± 0.6
*Klebsiella* species (10)	10/10	17.2 ± 0.9
*Proteus mirabilis* (1)	1/1	18.6
*Pseudomonas aeruginosa* (4)	4/4	18.9 ± 1.0
*Serratia marcescens* (2)	2/2	18.0 ± 1.1
*Staphylococcus aureus* (12)	12/12	19.8 ± 0.3
*Streptococcus* species (4)^†^	4/4	17.2 ± 4.3
Total (61)	60/61	

* *Not included in the target species panel*.

† *Including isolates identified as S. epidermidis (among coagulase-negative staphylococci) or S. gallolyticus, S. gordonii, S. oralis, and S. parasanguinis (among Streptococcus species)*.

**Table 3:** Results for 26 positive blood culture broth (PBCB) samples with polymicrobial growth tested by the MicrobScan assay.

**PBCB testing**				**MicrobScan assay testing**			
**Species identified**	**Microbial concentration (CFU/ml)**			**Positive detection for indicated species**	**Cq for single detection**		
	**Species 1**	**Species 2**	**Species 3**		**Species 1**	**Species 2**	**Species 3**
*A. baumannii*/*P. aeruginosa*	1.3 ×10^8^	1.1 ×10^7^	–	*A. baumannii*	17.89	–	–
*A. baumanni*i/*P. aeruginosa*	2.0 ×10^8^	1.0 ×10^6^	–	*A. baumannii*	17.02	–	–
*A. baumannii*/*S. epidermidis*	1.2 ×10^8^	1.2 ×10^8^	–	*A. baumanni*i/*Staphylococcus* spp.	17.11	17.21	–
*A. baumannii*/*S. epidermidis*	1.7 ×10^8^	1.1 ×10^8^	–	*A. baumannii*/*Staphylococcus* spp.	17.50	17.98	–
*C. albicans*/*K. oxytoca*/*E. cloacae*	3.0 ×10^7^	1.5 ×10^6^	1.2 ×10^6^	*C. albicans*/*Klebsiella* spp./*E. cloacae* complex	18.46	22.42	23.71
*C. albicans*/*S. epidermidis*	1.2 ×10^8^	1.2 ×10^6^	–	*C. albicans*/*Staphylococcus* spp.	17.95	22.11	–
*C. parapsilosis*/*S. capitis*	1.4 ×10^7^	1.2 ×10^7^	–	*Candida* spp./*Staphylococcus* spp.	18.94	19.31	–
*E. faecalis*/*A. baumannii*	1.3 ×10^8^	3.0 ×10^6^	–	*E. faecalis*/*A. baumannii*	16.40	22.90	–
*E. faecalis*/*C. albicans*	1.2 ×10^8^	1.2 ×10^6^	–	*E. faecalis*/*C. albicans*	17.97	21.20	–
*E. faecalis*/*S. aureus*	1.3 ×10^9^	1.2 ×10^8^	–	*E. faecalis*/*S. aureus*	14.88	17.02	–
*E. faecium*/*K. oxytoca*	1.3 ×10^8^	1.5 ×10^7^	–	*E. faecium*/*Klebsiella* spp.	17.94	20.63	–
*E. faecium*/*S. aureus*/*S. capitis*	1.7 ×10^9^	1.9 ×10^8^	1.2 ×10^7^	*E. faecium*/*S. aureus*/*Staphylococcus* spp.	14.60	17.29	20.34
*E. coli*/*K. pneumoniae*/*P. aeruginosa*	1.4 ×10^8^	1.6 ×10^7^	1.2 ×10^5^	*Escherichia coli*/*Klebsiella* spp.	16.28	20.77	–
*K. pneumoniae*/*E. cloacae*	2.1 ×10^8^	1.4 ×10^6^	–	*Klebsiella* spp.	17.32	–	–
*K. pneumoniae*/*E. coli*	3.0 ×10^9^	1.7 ×10^5^	–	*Klebsiella* spp.	18.74	–	–
*K. pneumoniae*/*P. aeruginosa*	2.1 ×10^8^	1.9 ×10^7^	–	*Klebsiella* spp./*P. aeruginosa*	18.25	19.22	–
*K. pneumoniae*/*S. hominis*	2.1 ×10^8^	1.5 ×10^7^	–	*Klebsiella* spp./*Staphylococcus* spp.	18.66	19.02	–
*P. mirabilis*/*S. aureus*	1.3 ×10^8^	1.0 ×10^7^	–	*P. mirabilis*/*S. aureus*	18.07	20.27	–
*P. aeruginosa*/C. striatum^*^	1.1 ×10^8^	2.0 ×10^6^	–	*P. aeruginosa*	18.04	–	–
*S. aureus*/*P. aeruginosa*	2.8 ×10^7^	1.7 ×10^7^	–	*S. aureus*/*P. aeruginosa*	21.15	21.35	–
*S. aureus*/*S. salivarius*	1.3 ×10^8^	2.5 ×10^6^	–	*S. aureus*/*Streptococcus* spp.	18.56	23.38	–
*S. aureus*/*S. anginosus*	1.3 ×10^8^	1.8 ×10^7^	–	*S. aureus*/*Streptococcus* spp.	20.24	21.28	–
*S. epidermidis*/*A. baumannii*	1.5 ×10^8^	1.2 ×10^6^	–	*Staphylococcus* spp.	17.59	–	–
*S. epidermidis*/*S. aureus*	1.7 ×10^8^	2.0 ×10^7^	–	*Staphylococcus* spp./*S. aureus*	19.65	20.83	–
*S. hominis*/*S. mitis*	1.3 ×10^8^	1.2 ×10^6^	–	*Staphylococcus* spp.	17.72	–	–
*S. anginosus*/C. ramosum^*^	1.4 ×10^8^	1.5 ×10^8^	–	*Streptococcus* spp.	17.46	–	–

* *Not included in the target species panel*.

### Direct Detection of Microbial Pathogens From Blood Samples

We conducted a clinical evaluation of the MicrobScan assay using whole-blood samples from patients with a suspected BSI who were hospitalized at the Fondazione Policlinico Universitario A. Gemelli IRCCS of Rome, Italy, during September 2017–August 2018. To evaluate the diagnostic accuracy of the assay, we compared the MicrobScan assay results with those of the BCs simultaneously performed as a standard diagnostic method (Table 4). Of the 229 samples tested, four samples were positive in culture for microorganisms not targeted by the MicrobScan assay (i.e., *Morganella morganii, N. meningitidis, Bacteroides thetaiotaomicron*, and *Listeria monocytogenes*). Overall, sensitivity and specificity were 72.1 and 86.6%, respectively. The remaining 225 samples (133 from adult patients and 92 from pediatric patients) yielded 56 organism detections (52 single and 4 multiple) by the assay, whereas BCs yielded 39 (35 monomicrobial and 4 polymicrobial). The polymicrobial BCs consisted of three cultures with two microorganisms and one culture with three microorganisms. The MicrobScan assay matched 31 of the 39 positive detections obtained by culture (79.5% agreement) and detected an additional 25 microorganisms in culture-negative samples (15 from pediatric patients and 10 from adult patients) (Table 4). The MicrobScan assay-positive, culture-negative detections regarded species such as *E. coli* (*N* = 8), *S. aureus* (*N* = 6), and *S. pneumoniae* (*N* = 4) in most cases, or species such as *C. glabrata* (*N* = 1) and *E. aerogenes* (*N* = 1). In all six samples with *mecA*-positive microorganism detection (4 *S. aureus* and 2 *S. epidermidis*), the results from the MicrobScan assay were concordant with the results obtained by the laboratory reference method. To support the MicrobScan assay-positive, culture-negative detection results, we used “clinical indications” of infection, which consisted of detecting the same microorganisms and identifying them as causal pathogens in concomitant analyses of sterile fluids other than blood, respiratory tract fluids or aspirates, and/or urinary tract samples from the same patients. By applying this criterion, the sensitivity and specificity increased to 86.4 and 97.0%, respectively, suggesting that the discordant results for 20 MicrobScan assay-positive/BC-negative samples (13 from pediatric patients and 7 from adult patients) were true positives (Table 4). Notably, 11 (84.6%) of 13 and 4 (57.1%) of 7, accounting for a total of 75.0% (15/20), of the patients were receiving antimicrobial therapy. Overall, 13 MicrobScan assay results remained discordant, five of which represented overdetection (2 *E. coli*, 1 *E. faecalis*, 1 *C. albicans*, and 1 *C. glabrata*) and eight missed detections (2 *E. faecalis*, 1 *K. pneumoniae*, 1 *S. aureus*, 1 *S. parasanguinis*, and 3 *Staphylococcus* species other than *S. aureus*). In addition to *S. parasanguinis* and one *E. faecalis* grown in pediatric bottles, the missed microorganisms included three coagulase-negative staphylococci, which were likely contaminants, and one *E. faecalis* (vancomycin susceptible), which was detected after 47.19 h of incubation and for which the clinical relevance could not be determined from the patient's clinical course. Two other microorganisms, one *K. pneumoniae* (carbapenem susceptible) and one *S. aureus* (methicillin susceptible), represented missed detections that were not explainable as contaminants or not clinically relevant. Finally, the mean (± SD) times to positive results by the MicrobScan assay and the BC method (including species identification) were 5.3 ± 0.2 h and 17.8 ± 12.1 h for bacterial species and 5.3 ± 0.2 h and 22.7 ± 10.1 h for *Candida* species, respectively (*P* < 0.001, for all comparisons). The mean (± SD) times to negative results by the MicrobScan assay and the BC method were 5.1 ± 0.1 h and 120.0 ± 0.0 h, respectively (*P* < 0.001).

**Table 4:** Performance of the MicrobScan assay in whole-blood samples from patients with suspected bloodstream infections.

	**MicrobScan assay results evaluated according to the criterion of**	
	**Blood culture**	**Clinically and/or laboratory-documented infection**
Total blood samples tested (*N* = 225) Matched positives, *n*	31	51
Matched negatives, *n*	161	161
MicrobScan assay overdetection, *n*	25	5
MicrobScan assay missed detections, *n*	8	8
Overall agreement, %	85.3	94.2
Sensitivity, %	79.5	86.4
Specificity, %	86.6	97.0
PPV, %	55.3	91.1
NPV, %	95.3	95.3

*Data from either blood culture testing or clinical and/or laboratory findings were used to evaluate the MicrobScan assay results*.

### Clinical Value Assessment

We assessed the MicrobScan assay from a strictly clinical standpoint by reviewing the records of the 31 BSI patients with positive detections by both the MicrobScan assay and the BC method. Based on the BC results, the physicians decided to change the antimicrobial therapy initiated at the BSI onset in 20 (64.5%) patients [mean (± SD) time to change, 21.3 ± 13.2 h]. Five patients had their antimicrobial therapy changed the same day (9.8 h ± 5.0 h), and 15 had their antimicrobial therapy changed on the subsequent days (25.1 ± 12.9 h) of BC collection. The initial antimicrobial therapy was escalated in 12 patients and de-escalated in eight patients. In the remaining 11 (35.5%) patients, the initial antimicrobial therapy was unchanged. Based on the MicrobScan assay results, the physicians could have escalated or de-escalated the antimicrobial therapy initiated at the BSI onset in the 20 patients with a mean (± SD) time of 11.6 ± 5.2 h. Furthermore, we assessed the impact of prior antimicrobial use on the MicrobScan assay performance in comparison with the BC method for all 225 cases (88 with and 137 without antimicrobials) studied. The subgroup analysis of the BC results showed that prior antimicrobial use was significantly associated with culture-negative but MicrobScan-positive cases compared with cases in which both culture and MicrobScan results were negative [72.0% (18/25) and 37.3% (60/161), respectively; *P* < 0.002]. Consistently, the subgroup analysis of the MicrobScan results showed that a previous antimicrobial use was significantly associated with MicrobScan-positive but culture-negative cases compared with cases in which both culture and MicrobScan results were positive [72.0% (18/25) and 29.0% (9/31), *P* < 0.003].

## Discussion

We show that the MicrobScan assay is capable of reliably identifying bacterial and fungal (*Candida*) pathogens directly in whole-blood samples from patients with a suspected BSI. The assay combines automated sample preparation with multiplex qPCR for selected targets within single-reaction wells, and detects 16 bacteria and 4 fungi as well as 3 antibiotic resistance determinants in ~5 h. Interestingly, in 31 (79.5%) of the 39 culture-documented BSI cases with concordant positive MicrobScan assay results, 20 patients could have benefited from timely appropriate antimicrobial therapy. In these patients, physicians changed the initial antimicrobial treatment at 21.3 ± 13.2 h following the notification of a BC result. These changes could have occurred 9.7 ± 8.0 h earlier in the case of a MicrobScan assay result notification. Nevertheless, assay failures occurred in eight of 39 (20.5%) samples, which is of concern. We hypothesized possible reasons for this lack of success, such as the uneven distribution of bacterial/*Candida* species within clinical samples or the errors relating to the sensitivity and specificity of qPCRs. Clinically appropriate management of sepsis and septic shock includes performing BCs before initiating antimicrobial therapy, with at least two BC pairs (using both aerobic and anaerobic bottles) collected per BSI episode (Rhodes et al., 2017). Among the eight cases with MicrobScan assay-negative results, we detected microorganisms (all in monomicrobial growth) in only one of the BC pairs obtained from the patients. Considering the clinical significance of four of these organisms questionable, the rate of false-negative results by the assay decreased to 10.2% (4/39), which is of less concern.

We sought to rule out any impairment in the accurate quantification of multiple targets in a single reaction by demonstrating that the assay efficiency and the LOD were the same as when the reactions ran in a singleplex manner. This issue is particularly crucial when amplifying targets of low relative abundance together with targets of high relative abundance (Bustin et al., 2009). We tested the MicrobScan assay on polymicrobial PBCB samples before blindly using it directly on whole-blood samples. Expectedly, the qPCR detection efficiency was good in all 24 of the evaluable 26 polymicrobial PBCBs. Two PBCBs grew, as a second microorganism, species (i.e., *C. striatum* and *C. ramosum*) not included in the target species panel of the MicrobScan assay. Failure to detect the second microorganism occurred in six samples and, surprisingly, two of undetected microorganisms were *P. aeruginosa*. This might be attributed to quantitative differences between the two microorganisms present in the same PBCB sample, especially when the two microorganisms had to be detected in the same reaction. Nevertheless, it is improbable that such high concentrations of qPCR template represent the microbial density levels in infected patient's blood samples before their “amplification” in culture, as in PBCBs. The microbial density in blood is generally very low, particularly for cases of invasive candidiasis that encompass candidemia and deep-seated candidiasis (Clancy and Nguyen, 2013). Indeed, the LOD for BCs is comparable to that of PCR-based methods, at least in invasive *Candida* infections (Arvanitis et al., 2014).

Using BCs as a comparator may not be appropriate due to limitations including sensitivity. We know that a number of factors can influence the diagnostic accuracy of BCs, and antimicrobial therapy prior to blood sampling is one such factor (among others) (Lamy et al., 2018). However, PCR-based detection of bacteria and fungi in culture-negative clinical samples, especially obtained from patients on antimicrobial therapy, continues to create a clinical interpretation challenge (Farrell et al., 2013). MicrobScan assay-positive/culture-negative results may promote the inappropriate antimicrobial treatment of patients who are unlikely to have bacteremia or candidemia, which would inadvertently lead to unintended consequences for institutional efforts to optimize antimicrobial prescription practices (Murri et al., 2018). Among the 225 patients studied, 88 (39.1%) were under antimicrobial treatment at the time a blood sample was collected for testing with the MicrobScan assay. We showed that prior antimicrobial exposure did not adversely affect the performance of the MicrobScan assay. Eighteen (23.1%) of 78 patients with a negative BC result and nine (90.0%) of 10 patients with a positive BC result had positive detection results in the MicrobScan assay.

We performed an in-depth culture-based analysis of the 25 patient cases where the MicrobScan assay identified additional microorganisms. We found that 20 of 25 microorganisms were isolated and identified as causal pathogens from the primary cultures of clinical specimens obtained from the same patients almost concomitantly with the blood samples for MicrobScan assay testing. In all 20 cases, the microorganism was the same, thus reflecting the source of infection, and interestingly, 15 (75.0%) of the 20 patients were receiving antimicrobial therapy. More interestingly, the MicrobScan assay mirrors what seen with the T2 magnetic resonance-based method, from which it differs technologically (T2 Biosystems, Lexington, Massachusetts, USA; www.t2biosystems.com). A recent multicenter study by Nguyen et al. (2019) showed that 13.0% of the patients studied had BC and T2Bacteria results positive for the six T2Bacteria-targeted bacterial species. Among negative BCs with a positive T2Bacteria result, 60.0% of them were associated with probable or possible BSIs (Nguyen et al., 2019). In our previous evaluation of the T2Bacteria^®^ Panel (De Angelis et al., 2018), we found 89.0% sensitivity and 98.0% specificity among patients with positive BCs for the 6 bacterial species or fulfilling the criteria for infection (named CI, but similar to those specified above). Consistent with the present study, T2 Bacteria-positive/BC-negative results were significantly more likely to occur among patients receiving antimicrobial therapy (*P* < 0.001). Taken together, these findings show that the MicrobScan assay may be particularly worth using in conjunction with cultures and during antimicrobial therapy.

In summary, the MicrobScan assay has the potential to rapidly exclude and identify diverse bacterial and *Candida* BSIs, including cases that the standard BCs may not detect. The spectrum of molecular targets is restricted to common infecting pathogens and antimicrobial resistance-associated genes. However, we noticed that the MicrobScan assay targets cover ~92% of the BSIs diagnosed in our clinical microbiology laboratory (Fiori et al., 2016), which yet excludes clinically relevant species such as *N. meningitidis* or *H. influenzae*. Theoretically, small laboratories without experience in molecular detection platforms and techniques may be suitable users. In practice, it is mandatory that clinical microbiologists interpret the MicrobScan assay results in the context of clinical and other laboratory findings, as with current culture-based identifications. Thus, we believe that large laboratories can better supply both expertise and additional information. Carefully designed clinical studies are therefore necessary to establish the role that the MicrobScan assay may play in the future microbiology laboratory.

## Data Availability Statement

The datasets generated for this study are available on request to the corresponding author.

## Ethics Statement

The studies involving human participants were reviewed by the Ethics Committee of the Fondazione Policlinico Universitario A. Gemelli IRCCS of Rome (Italy), which approved the study protocol (Approval No. 0023266/17) and required informed consent be obtained from all participants and/or their legal guardian(s). All the methods described in the study were in accordance with the Declaration of Helsinki and national and international standards. The patients/participants provided their written informed consent to participate in this study.

## Author Contributions

FL performed experiments for the clinical validation of the MicrobScan assay. BP, FT, and MS designed the study and planned the experiments. GD and GM collected clinical samples and performed the analysis of the data. FM, FC, AP, and PF performed experiments for the creation and establishment of the MicrobScan assay. TS and MS supervised the study. BP wrote the first draft of the article and prepared the figures and tables. All authors wrote and revised the manuscript and approved the final version of the manuscript.

### Conflict of Interest

FM and FC are employed by the company Nurex S.r.l. The remaining authors declare that the research was conducted in the absence of any commercial or financial relationships that could be construed as a potential conflict of interest.

## References

[B1] Andes D. R. Safdar N. Baddley J. W. Alexander B. Brumble L. Freifeld A.. (2016). The epidemiology and outcomes of invasive *Candida* infections among organ transplant recipients in the United States: results of the Transplant-Associated Infection Surveillance Network (TRANSNET). Transpl. Infect. Dis. 18, 921–931. 10.1111/tid.12613 27643395

[B2] Arvanitis M. Anagnostou T. Fuchs B. B. Caliendo A. M. Mylonakis E. (2014). Molecular and nonmolecular diagnostic methods for invasive fungal infections. Clin. Microbiol. Rev. 27, 490–526. 10.1128/CMR.00091-13 24982319 PMC4135902

[B3] Banerjee R. Özenci V. Patel R. (2016). Individualized approaches are needed for optimized blood cultures. Clin. Infect. Dis. 63, 1332–1339. 10.1093/cid/ciw573 27558570 PMC5091349

[B4] Baron E. J. Miller J. M. Weinstein M. P. Richter S. S. Gilligan P. H. Thomson R. B. Jr (2013). A guide to utilization of the microbiology laboratory for diagnosis of infectious diseases: 2013 recommendations by the Infectious Diseases Society of America (IDSA) and the American Society for Microbiology (ASM). Clin. Infect. Dis. 57, e22–e121. 10.1093/cid/cit441 23845951 PMC3719886

[B5] Blauwkamp T. A. Thair S. Rosen M. J. Blair L. Lindner M. S. Vilfan I. D.. (2019). Analytical and clinical validation of a microbial cell-free DNA sequencing test for infectious disease. Nat. Microbiol. 4, 663–674. 10.1038/s41564-018-0349-6 30742071

[B6] Bustin S. A. Benes V. Garson J. A. Hellemans J. Huggett J. Kubista M.. (2009). The MIQE guidelines: minimum information for publication of quantitative real-time PCR experiments. Clin. Chem. 55, 611–622. 10.1373/clinchem.2008.112797 19246619

[B7] Clancy C. J. Nguyen M. H. (2013). Finding the “missing 50%” of invasive candidiasis: how nonculture diagnostics will improve understanding of disease spectrum and transform patient care. Clin. Infect. Dis. 56, 1284–1292. 10.1093/cid/cit006 23315320

[B8] De Angelis G. Posteraro B. De Carolis E. Menchinelli G. Franceschi F. Tumbarello M.. (2018). T2Bacteria magnetic resonance assay for the rapid detection of ESKAPEc pathogens directly in whole blood. J. Antimicrob. Chemother. 73(Suppl 4), iv20–iv26. 10.1093/jac/dky049 29608753

[B9] De Gregorio E. Roscetto E. Iula V. D. Martinucci M. Zarrilli R. Di Nocera P. P.. (2015). Development of a real-time PCR assay for the rapid detection of *Acinetobacter baumannii* from whole blood samples. N. Microbiol. 38, 251–257. 25938750

[B10] Dubourg G. Raoult D. (2016). Emerging methodologies for pathogen identification in positive blood culture testing. Exp. Rev. Mol. Diagn. 16, 97–111. 10.1586/14737159.2016.1112274 26559655

[B11] Farrell J. J. Sampath R. Ecker D. J. Bonomo R. A. (2013). “Salvage microbiology”: detection of bacteria directly from clinical specimens following initiation of antimicrobial treatment. PLoS ONE 8:e66349 10.1371/journal.pone.0066349 23825537 PMC3692526

[B12] Fiori B. D'Inzeo T. Di Florio V. De Maio F. De Angelis G. Giaquinto A.. (2014). Performance of two resin-containing blood culture media in detection of bloodstream infections and in direct matrix-assisted laser desorption ionization-time of flight mass spectrometry (MALDI-TOF MS) broth assays for isolate identification: clinical comparison of the BacT/Alert Plus and Bactec Plus systems. J. Clin. Microbiol. 52, 3558–3567. 10.1128/JCM.01171-14 25031441 PMC4187791

[B13] Fiori B. D'Inzeo T. Giaquinto A. Menchinelli G. Liotti F. M. de Maio F.. (2016). Optimized use of the MALDI biotyper system and the filmArray BCID panel for direct identification of microbial pathogens from positive blood cultures. J. Clin. Microbiol. 54, 576–584. 10.1128/JCM.02590-15 26677254 PMC4767970

[B14] Fleischmann C. Scherag A. Adhikari N. K. Hartog C. S. Tsaganos T. Schlattmann P.. (2016). Assessment of global incidence and mortality of hospital-treated sepsis. Current estimates and limitations. Am. J. Respir. Crit. Care Med. 193, 259–272. 10.1164/rccm.201504-0781OC 26414292

[B15] Fukumoto H. Sato Y. Hasegawa H. Saeki H. Katano H. (2015). Development of a new real-time PCR system for simultaneous detection of bacteria and fungi in pathological samples. Int. J. Clin. Exp. Pathol. 8, 15479–15488. 26823918 PMC4713704

[B16] Gadsby N. J. McHugh M. P. Russell C. D. Mark H. Conway Morris A. Laurenson I. F.. (2015). Development of two real-time multiplex PCR assays for the detection and quantification of eight key bacterial pathogens in lower respiratory tract infections. Clin. Microbiol. Infect. 21, 788.e1–788.e13 10.1016/j.cmi.2015.05.004 25980353 PMC4509705

[B17] Ginn A. N. Halliday C. L. Douglas A. P. Chen S. C. (2017). PCR-based tests for the early diagnosis of sepsis. Where do we stand? Curr. Opin. Infect. Dis. 30, 565–572. 10.1097/QCO.0000000000000407 29095722

[B18] Goto M. Al-Hasan M. N. (2013). Overall burden of bloodstream infection and nosocomial bloodstream infection in North America and Europe. Clin. Microbiol. Infect. 19, 501–509. 10.1111/1469-0691.12195 23473333

[B19] Kadri S. S. Rhee C. Strich J. R. Morales M. K. Hohmann S. Menchaca J.. (2017). Estimating ten-year trends in septic shock incidence and mortality in United States academic medical centers using clinical data. Chest 151, 278–285. 10.1016/j.chest.2016.07.010 27452768 PMC5310115

[B20] Lamy B. Dargère S. Arendrup M. C. Parienti J. J. Tattevin P. (2016). How to optimize the use of blood cultures for the diagnosis of bloodstream infections? A state-of-the art. Front. Microbiol. 7:697. 10.3389/fmicb.2016.00697 27242721 PMC4863885

[B21] Lamy B. Ferroni A. Henning C. Cattoen C. Laudat P. (2018). How to: accreditation of blood cultures' proceedings. A clinical microbiology approach for adding value to patient care. Clin. Microbiol. Infect. 24, 933–934. 10.1016/j.cmi.2018.02.025 29410246

[B22] Metwally L. Hogg G. Coyle P. V. Hay R. J. Hedderwick S. McCloskey B.. (2007). Rapid differentiation between fluconazole-sensitive and -resistant species of *Candida* directly from positive blood-culture bottles by real-time PCR. J. Med. Microbiol. 56, 964–970. 10.1099/jmm.0.47149-0 17577063

[B23] Murri R. Taccari F. Spanu T. D'Inzeo T. Mastrorosa I. Giovannenze F.. (2018). A 72-h intervention for improvement of the rate of optimal antibiotic therapy in patients with bloodstream infections. Eur. J. Clin. Microbiol. Infect. Dis. 37, 167–173. 10.1007/s10096-017-3117-2 29052092

[B24] Nguyen M. H. Clancy C. J. Pasculle A. W. Pappas P. G. Alangaden G. Pankey G. A. (2019). Performance of the T2Bacteria panel for diagnosing bloodstream infections: a diagnostic accuracy study. Ann. Intern. Med. 17, 845–852. 10.7326/M18-2772 31083728

[B25] Papaparaskevas J. Mela V. Houhoula D. P. Pantazatou A. Petrikkos G. L. Tsakris A. (2013). Comparative evaluation of conventional and real-time PCR assays for detecting *Bacteroides fragilis* in clinical samples. J. Clin. Microbiol. 51, 1593–1595. 10.1128/JCM.00449-13 23447634 PMC3647933

[B26] Peker N. Couto N. Sinha B. Rossen J. W. (2018). Diagnosis of bloodstream infections from positive blood cultures and directly from blood samples: recent developments in molecular approaches. Clin. Microbiol. Infect. 24, 944–955. 10.1016/j.cmi.2018.05.007 29787889

[B27] Pfeiffer C. D. Samsa G. P. Schell W. A. Reller L. B. Perfect J. R. Alexander B. D. (2011). Quantitation of *Candida* CFU in initial positive blood cultures. J. Clin. Microbiol. 49, 2879–2883. 10.1128/JCM.00609-11 21677065 PMC3147732

[B28] Pliakos E. E. Andreatos N. Shehadeh F. Ziakas P. D. Mylonakis E. (2018). The cost-effectiveness of rapid diagnostic testing for the diagnosis of bloodstream infections with or without antimicrobial stewardship. Clin. Microbiol. Rev. 31, e00095–e00017. 10.1128/CMR.00095-17 29848775 PMC6056844

[B29] Pruinelli L. Westra B. L. Yadav P. Hoff A. Steinbach M. Kumar V.. (2018). Delay within the 3-hour surviving sepsis campaign guideline on mortality for patients with severe sepsis and septic shock. Crit. Care Med. 46, 500–505. 10.1097/CCM.0000000000002949 29298189 PMC5851815

[B30] Rhodes A. Evans L. E. Alhazzani W. Levy M. M. Antonelli M. Ferrer R.. (2017). Surviving sepsis campaign: international guidelines for management of sepsis and septic shock: 2016. Intensive Care Med. 43, 304–377. 10.1007/s00134-017-4683-6 28101605

[B31] Seymour C. W. Gesten F. Prescott H. C. Friedrich M. E. Iwashyna T. J. Phillips G. S.. (2017). Time to treatment and mortality during mandated emergency care for sepsis. N. Engl. J. Med. 376, 2235–2244. 10.1056/NEJMoa1703058 28528569 PMC5538258

[B32] Thomas L. C. Gidding H. F. Ginn A. N. Olma T. Iredell J. (2007). Development of a real-time *Staphylococcus aureus* and MRSA (SAM-) PCR for routine blood culture. J. Microbiol. Methods 68, 296–302. 10.1016/j.mimet.2006.09.003 17046087

[B33] Timbrook T. T. Morton J. B. McConeghy K. W. Caffrey A. R. Mylonakis E. LaPlante K. L. (2017). The effect of molecular rapid diagnostic testing on clinical outcomes in bloodstream infections: a systematic review and meta-analysis. Clin. Infect. Dis. 64, 15–23. 10.1093/cid/ciw649 27678085

[B34] van den Brand M. Peters R. P. H. Catsburg A. Rubenjan A. Broeke F. J. van den Dungen F. A. M.. (2014). Development of a multiplex real-time PCR assay for the rapid diagnosis of neonatal late onset sepsis. J. Microbiol. Methods 106, 8–15. 10.1016/j.mimet.2014.07.034 25102109

[B35] van der Zee A. Roorda L. Bosman G. Ossewaarde J. M. (2016). Molecular diagnosis of urinary tract infectionsby semi-quantitative detection of uropathogens in a routine clinical hospital setting. PLoS ONE 11:e0150755. 10.1371/journal.pone.0150755 26954694 PMC4783162

[B36] Whiles B. B. Deis A. S. Simpson S. Q. (2017). Increased time to initial antimicrobial administration is associated with progression to septic shock in severe sepsis patients. Crit. Care Med. 45, 623–629. 10.1097/CCM.0000000000002262 28169944 PMC5374449

[B37] Yagupsky P. Nolte F. S. (1990). Quantitative aspects of septicemia. Clin. Microbiol. Rev. 3, 269–279. 10.1128/CMR.3.3.269 2200606 PMC358159

[B38] Zasowski E. J. Claeys K. C. Lagnf A. M. Davis S. L. Rybak M. J. (2016). Time is of the essence: the impact of delayed antibiotic therapy on patient outcomes in hospital-onset enterococcal bloodstream infections. Clin. Infect. Dis. 62, 1242–1250. 10.1093/cid/ciw110 26945013 PMC4845789

[B39] Zheng F. Sun J. Cheng C. Rui Y. (2013). The establishment of a duplex real-time PCR assay for rapid and simultaneous detection of *bla*_NDM_ and *bla*_KPC_ genes in bacteria. Ann. Clin. Microbiol. Antimicrob. 12:30. 10.1186/1476-0711-12-30 24143953 PMC3816589

