# Supplementary material for: Development of a Multiplex PCR Platform for the Rapid Detection of Bacteria, Antibiotic Resistance, and Candida in Human Blood Samples
Source: Front Cell Infect Microbiol. 2019 Nov 13;9:389. doi: 10.3389/fcimb.2019.00389 (PMC6863929; doi:10.3389/fcimb.2019.00389)
Supplement: Supplementary file 1 [file Table_1.docx]

Supplementary material

**TABLE S1.** Limit of detection (LOD) estimation for all gram-negative (bacteria), gram-positive (enterococci, staphylococci, and streptococci), and *Candida* species targeted by the MicrobScan assay in either sterile buffer (phosphate-buffered saline) or otherwise negative human whole blood.

| **Spiked microorganism** | **95% LOD (confidence interval) in phosphate-buffered saline** | **PCR efficiency** | **95% LOD (confidence interval) in whole blood** | **PCR efficiency** |
| --- | --- | --- | --- | --- |
| Gram-negatives |  |  |  |  |
| *A. baumannii* | 13.5 (12.2–14.8) | >98% | 12.4 (9.8–26.9) | >98% |
| B. fragilis | 11.2 (8.6–38.2) | >99% | 10.6 (9.9–11.3) | >99% |
| *E. aerogenes* | 14.8 (11.0–56.8) | >99% | 13.5 (12.2–14.8) | >99% |
| *E. cloacae* | 14.5 (11.3–40.6) | >99% | 14.5 (11.3–40.6) | >98% |
| E. coli | 11.2 (10.5–11.9) | >98% | 10.9 (10.3–11.6) | >98% |
| *K. oxytoca* | 15.5 (11.3–70.4) | >99% | 14.8 (11.0–56.8) | >99% |
| K. pneumoniae | 14.5 (11.3–40.6) | >99% | 14.5 (11.3–40.6) | >99% |
| *P. aeruginosa* | 12.4 (9.8–26.9) | >99% | 12.4 (9.8–26.9) | >99% |
| *P. mirabilis* | 13.5 (9.9–13.8) | >98% | 12.7 (11.7–13.6) | >98% |
| *S. marcescens* | 11.9 (9.9–19.7) | >99% | 11.2 (10.5–11.9) | >98% |
| Gram-positives |  |  |  |  |
| *E. faecalis* | 17.1 (12.4–54.8) | >97% | 16.2 (12.2–46.3) | >97% |
| *E. faecium* | 16.4 (12.2–46.3) | >98% | 16.9 (12.9–38.7) | >99% |
| *St. aureus* | 17.1 (12.4–54.8) | >98% | 18.6 (14.2–36.9) | >98% |
| *St. epidermidis* | 16.9 (12.9–38.7) | >98% | 18.6 (14.2–36.9) | >98% |
| *Str. hominis* | 18.9 (14.8–32.6) | >98% | 18.9 (14.8–32.6) | >99% |
| *Str. agalactiae* | 20.0 (14.9–45.7) | >99% | 20.2 (15.5–37.9) | >99% |
| *Str. pneumoniae* | 21.6 (16.2–45.8) | >98% | 20.3 (13.9–50.7) | >98% |
| *Str. pyogenes* | 19.7 (14.0–63.3) | >99% | 20.8 (16.1–38.0) | >99% |
| Fungi |  |  |  |  |
| *C. albicans* | 15.9 (12.2–41.1) | >99% | 15.9 (12.2–41.1) | >98% |
| *C. glabrata* | 18.9 (14.8–32.6) | >97% | 18.6 (14.2–36.9) | >99% |
| *C. krusei* | 19.1 (14.1–45.1) | >98% | 18.9 (14.8–32.6) | >99% |
| *C. orthopsilosis* | 15.9 (12.2–41.1) | >99% | 14.8 (11.0–56.8) | >99% |
| *C. parapsilosis* | 14.5 (11.3–40.6) | >99% | 15.5 (11.3–70.4) | >98% |
| *C. tropicalis* | 14.8 (11.0–56.8) | >98% | 14.5 (11.3–40.6) | >98% |

*The 95% LOD is the density (CFU/ml) at which the target microorganism is detected 95% of the time. The efficiency of the relative singleplex PCRs performed for each organism is also shown.*

**TABLE S2.** Specificity of the MicrobScan assay assessed by testing reference and/or clinical isolates from bacterial and *Candida* species in both sterile buffer and whole blood matrices.

| Species (no. of isolates tested) | **Reaction 1**  *S. aureus* and **Staphylococcus* spp. | **Reaction 2**  *E. aerogenes*, *E. cloacae* complex, and **Klebsiella* spp. | **Reaction 3**  *E. faecalis*, *E. faecium*, and **Streptococcus* spp. | **Reaction 4**  *A. baumannii*, *E. coli*, *P. aeruginosa*, and *P. mirabilis* | **Reaction 5**  *B. fragilis*, *S. marcescens*, *S. pyogenes*, and *S. pneumoniae* | **Reaction 6**  *C. albicans*/*C. glabrata*, *C. krusei*, and **Candida* spp. | **Reaction 7**  *bla*_KPC_, *mecA*, and *vanA*/*vanB* |
| --- | --- | --- | --- | --- | --- | --- | --- |
| *A. baumannii* (10) | 0 | 0 | 0 | 10 | 0 | 0 | 0 |
| *B. fragilis* (10) | 0 | 0 | 0 | 0 | 10 | 0 | 0 |
| *C. albicans* (10) | 0 | 0 | 0 | 0 | 0 | 10 | 0 |
| *C. glabrata* (10) | 0 | 0 | 0 | 0 | 0 | 10 | 0 |
| *C. krusei* (10) | 0 | 0 | 0 | 0 | 0 | 10 | 0 |
| *C. orthopsilosis* (10) | 0 | 0 | 0 | 0 | 0 | 10 | 0 |
| *C. parapsilosis* (10) | 0 | 0 | 0 | 0 | 0 | 10 | 0 |
| *C. tropicalis* (10) | 0 | 0 | 0 | 0 | 0 | 10 | 0 |
| *E. aerogenes* (10) | 0 | 10 | 0 | 0 | 0 | 0 | 0 |
| *E. cloacae* (10) | 0 | 10 | 0 | 0 | 0 | 0 | 0 |
| *E. coli* (24) | 0 | 0 | 0 | 24 | 0 | 0 | 0 |
| *E. faecalis* (14) | 0 | 0 | 14 | 0 | 0 | 0 | 0 |
| *E. faecium* (10) | 0 | 0 | 10 | 0 | 0 | 0 | 0 |
| *K. oxytoca* (10) | 0 | 10 | 0 | 0 | 0 | 0 | 0 |
| *K. ozaenae* (10) | 0 | 10 | 0 | 0 | 0 | 0 | 0 |
| *K. pneumoniae* (16) | 0 | 10 | 0 | 0 | 0 | 0 | 0 |
| *P. aeruginosa* (11) | 0 | 0 | 0 | 11 | 0 | 0 | 0 |
| *P. mirabilis* (10) | 0 | 0 | 0 | 10 | 0 | 0 | 0 |
| *S. marcescens* (10) | 0 | 0 | 0 | 0 | 10 | 0 | 0 |
| *S. aureus* (25) | 25 | 0 | 0 | 0 | 0 | 0 | 0 |
| *S. epidermidis* (18) | 18 | 0 | 0 | 0 | 0 | 0 | 0 |
| *S. haemolyticus* (10) | 10 | 0 | 0 | 0 | 0 | 0 | 0 |
| *S. hominis* (10) | 10 | 0 | 0 | 0 | 0 | 0 | 0 |
| *S. agalactiae* (10) | 0 | 0 | 10 | 0 | 0 | 0 | 0 |
| *S. pyogenes* (10) | 0 | 0 | 0 | 0 | 10 | 0 | 0 |
| *S. salivarius* (10) | 0 | 0 | 10 | 0 | 0 | 0 | 0 |
| *S. parasanguinis* (10) | 0 | 0 | 10 | 0 | 0 | 0 | 0 |
| *S. pneumoniae* (10) | 0 | 0 | 10 | 0 | 10 | 0 | 0 |
| Nontargeted species† (10) | 0 | 0 | 0 | 0 | 0 | 0 | 0 |
| Total species (338) | – | – | – | – | – | – | – |
| *bla*_KPC_-positive *Escherichia* spp. (10) | 0 | 0 | 0 | 10 | 0 | 0 | 10 |
| *bla*_KPC_-positive *Klebsiella* spp. (10) | 0 | 10 | 0 | 0 | 0 | 0 | 10 |
| *mecA*-positive *Staphylococcus* spp. (10) | 10 | 0 | 0 | 0 | 0 | 0 | 10 |
| *vanA*/*vanB*-positive *Enterococcus* spp. (10) | 0 | 0 | 10 | 0 | 0 | 0 | 10 |

*No cross-reactivity was recorded because each test species, with or without any antibiotic resistance determinant (i.e., bla_KPC_, mecA, vanA/vanB), yielded a fluorescence signal(s) that only corresponded to the specific probe(s) in the multiplex PCR well(s). *Including targeted species such as Staphylococcus spp. other than S. aureus (e.g., S. epidermidis, S. haemolyticus, S. hominis, S. lugdunensis, and S. warneri); K. oxytoca, K. ozaenae, and K. pneumoniae; Streptococcus spp. other than S. pneumoniae or S. pyogenes (e.g., S. agalactiae, S. salivarius, and S. parasanguinis); and C. orthopsilosis, C. parapsilosis, and C. tropicalis. †Including nontargeted species such as Aspergillus fumigatus, Burkholderia cepacia, Corynebacterium striatum, Cryptococcus neoformans, Haemophilus influenzae, Legionella pneumophila, Moraxella catarrhalis, Neisseria meningitidis, Proteus vulgaris, and Stenotrophomonas maltophilia.*
